# Detection of benign granular cell tumor of the breast via ^18^F-PSMA-PET/CT in a patient with very high-risk prostate cancer: A case report

**DOI:** 10.1177/2050313X241275826

**Published:** 2024-08-31

**Authors:** Kamyar Ghabili, Rushi Rajyaguru, Alexandra De La Plante, Kristine L. Widders, Alison L. Chetlen, Angela I. Choe, Claudia J. Kasales

**Affiliations:** 1 Department of Radiology, Penn State Health Milton S. Hershey Medical Center, Hershey, PA, USA; 2 Department of Pathology, Penn State Health Milton S. Hershey Medical Center, Hershey, PA, USA; 3 Department of Surgery, Penn State Health Milton S. Hershey Medical Center, Hershey, PA, USA

**Keywords:** Breast, granular cell tumor, positron emission tomography, prostate cancer, prostate-specific membrane antigen

## Abstract

Incidental extra-prostatic prostate-specific membrane antigen (PSMA) uptake on initial staging positron emission tomography/computed tomography (PET/CT) scans poses diagnostic challenges, as it can be associated with various benign and malignant lesions. We present the case of a 68-year-old man with very high-risk prostate cancer who was incidentally discovered to have a benign granular cell tumor in the breast initially detected on PSMA-PET/CT. Imaging studies and biopsy were pivotal in the diagnosis, as the tumor’s appearance was concerning for breast carcinoma. Recognizing extra-prostatic PSMA uptake in the breast, particularly in patients with prostate cancer, is crucial for guiding appropriate management, accurately interpreting subsequent imaging findings, and assessing radiologic-pathologic correlation.

## Introduction

Incidental extra-prostatic prostate-specific membrane antigen (PSMA) uptake on the initial staging positron emission tomography/computed tomography scan (PET/CT) in patients with prostate cancer presents a challenge, as the differential diagnosis is broad.^1^ In breast tissue, PSMA avidity on PSMA-PET/CT imaging has been linked to various benign and malignant lesions, including fibroadenoma,^2^ pseudoangiomatous stromal hyperplasia (PASH),^3^ gynecomastia, and breast carcinoma.^4^ Characterization with diagnostic imaging and biopsy is often required.^5^ Rarely, abnormal PSMA uptake in the breast has been associated with prostate cancer metastasis.^6–8^ It is important to differentiate between breast metastasis from prostate cancer and primary male breast cancer, as hormonal therapy for prostate cancer is contraindicated in cases of breast cancer.^6^

Granular cell tumor of the breast is typically a benign neoplasm, although a few cases demonstrating malignant features have been reported in the literature.^9–11^ Initial presentation on mammography and ultrasound includes an irregular mass with imaging features resembling breast carcinoma.^10^ Therefore, tissue sampling is essential. Lack of fluorodeoxyglucose (FDG) avidity on PET/CT has also been proposed as a feature differentiating benign granular cell tumors from other breast malignancies.^12^ However, the imaging characteristics of benign granular cell tumors of the breast on PSMA-PET/CT have not been reported. We present a man with very high-risk prostate cancer who was incidentally found to have a benign granular cell tumor in the breast detected on PSMA-PET/CT.

## Case presentation

A 68-year-old white man with a past medical history of obesity, hyperlipidemia, hypertension, gout, diabetes mellitus, nephrolithiasis, and benign prostatic hyperplasia treated with dutasteride presented with an elevated serum prostate-specific antigen of 6.68 ng/mL in the absence of lower urinary tract symptoms. His family history was notable for breast cancer in his maternal grandmother at the age of 92. No suspicious nodules were palpated on digital rectal examination. Multiparametric magnetic resonance imaging (MRI) of the prostate showed a prostate imaging reporting and data system category five lesion in the right transition zone, prompting an MRI/transrectal ultrasound fusion biopsy of the prostate.^13^ The biopsy returned Gleason score 5 + 4 = 9 (grade group 5), consistent with stage IIIC disease or National Comprehensive Cancer Network (NCCN) very high-risk prostate cancer.^14,15^

**Figure 1.**
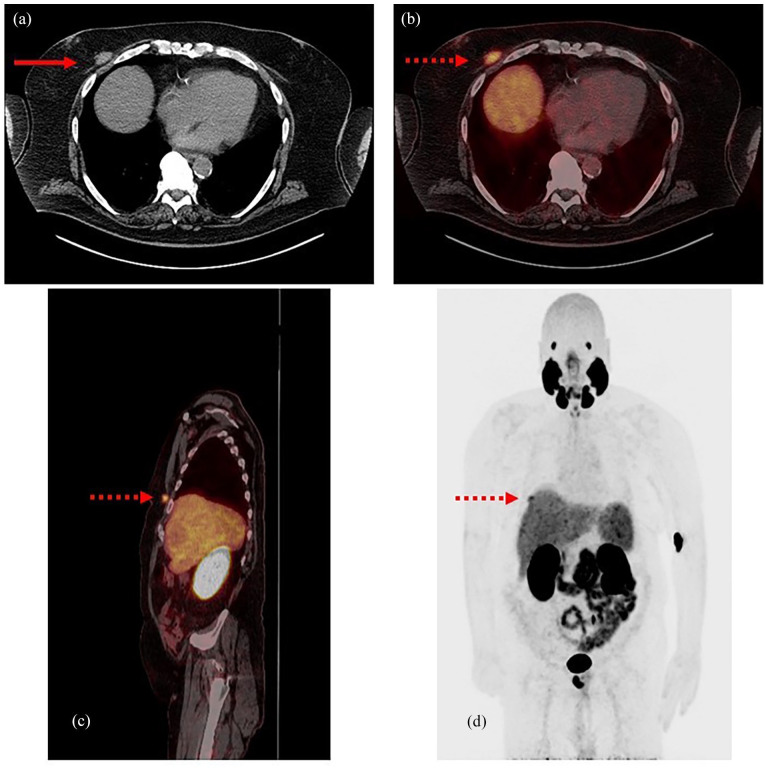
CT and ^18^F-PYLARIFY PSMA-PET images. (a) Noncontrast axial CT image displays a soft tissue mass along the right anterior chest wall abutting the pectoralis muscle (red arrow). Concordant abnormal PSMA radiotracer uptake (SUV_max_ 7.7) is depicted on axial (b) and sagittal fused PET and noncontrast CT (c) images, and on whole body maximum intensity projection image (d) (dashed red arrows). PSMA: prostate-specific membrane antigen; PET: positron emission tomography; CT: computed tomography.

Initial staging of the prostate cancer, 1 month after the diagnosis, was performed with ^18^F-PSMA-PET/CT, which demonstrated abnormal radiotracer activity within the prostate with a maximum standardized uptake value (SUV_max_) of 32.6. Another focus was noted along the right anterior chest wall (SUV_max_ 7.7) (Figure 1(a)–(d)), with no corresponding mass on physical examination of his breasts. No additional foci of abnormal radiotracer activity were noted. He denied breast trauma and was referred for evaluation in the breast clinic. The patient opted to proceed with external-beam radiation therapy, androgen deprivation therapy, and abiraterone for the management of his prostate cancer.

**Figure 2.**
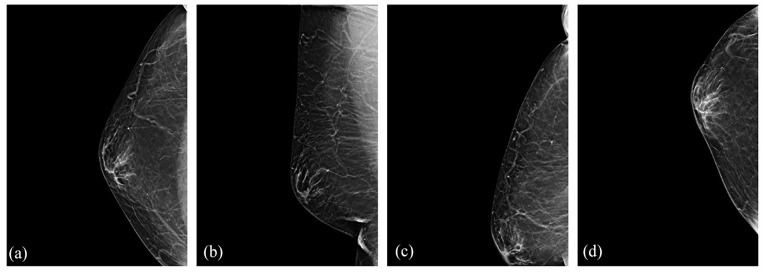
Right diagnostic mammogram including CC (a), MLO (b), XCCL (c), and XCCM; (d) views shows no significant masses, suspicious calcifications, or other abnormalities. CC: craniocaudal; MLO: mediolateral oblique; XCCL: exaggerated lateral CC; XCCM: exaggerated medial CC.

**Figure 3.**
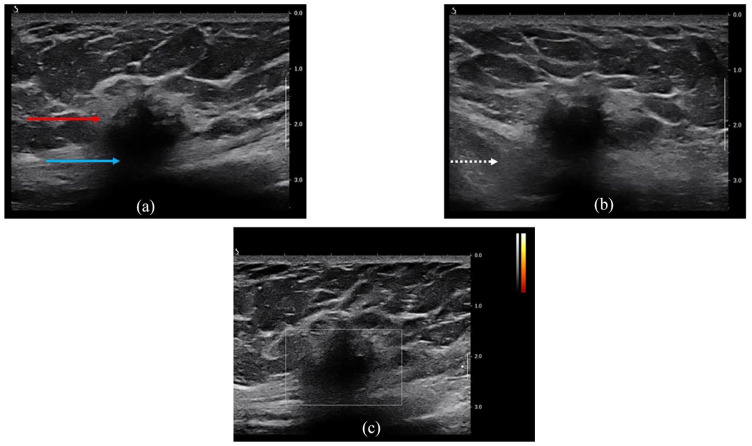
High-resolution real-time ultrasound of the right breast reveals a 1.5 × 2.1 × 1.8 cm irregular hypoechoic mass (red arrow) with spiculated margins and posterior shadowing (blue arrow) in the lower inner quadrant at 5 o’clock, located 3 cm from the nipple (a). The mass abuts the pectoralis musculature (dashed white arrow) (b). No internal vascularity was observed (c).

A diagnostic bilateral mammogram revealed no significant mass, suspicious calcifications, or other abnormalities in the right breast (Figure 2(a)–(d)). However, targeted ultrasound of the right breast identified a 1.5 × 2.1 × 1.8 cm irregular hypoechoic mass with spiculated margins and posterior shadowing in the lower inner quadrant at the 5 o’clock posterior depth, approximately 3 cm from the nipple (Figure 3(a)). The mass abutted the pectoralis muscle (Figure 3(b)). No internal vascularity was observed (Figure 3(c)), and no axillary adenopathy was detected on ultrasound. The lesion was characterized as suspicious, and a core needle biopsy was recommended.

**Figure 4.**
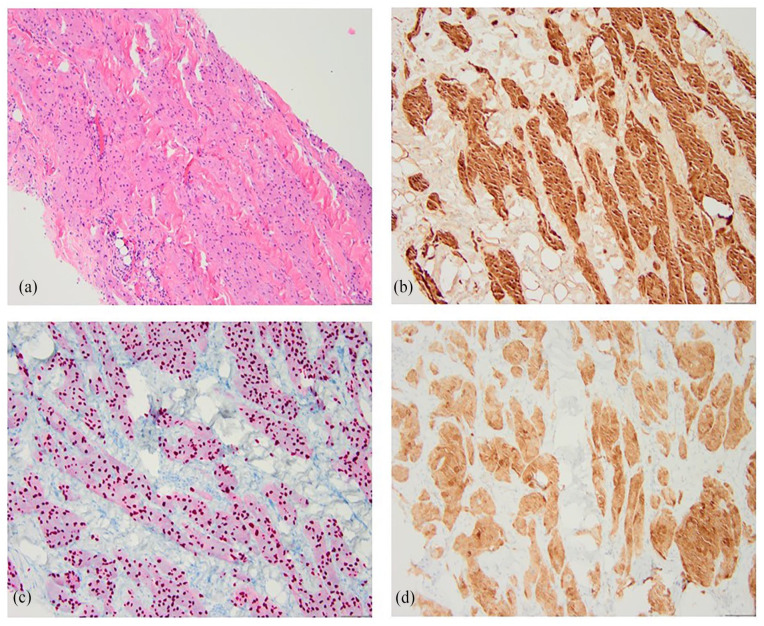
Hematoxylin and eosin staining of the biopsy specimen from the right breast mass depicts infiltrating aggregates of bland atypical cells with indistinct cell borders, eosinophilic, granular, abundant cytoplasm, and small nuclei (a). Immunohistochemical staining is positive for S-100 (b), SOX10 (c), and neuron-specific enolase (d). These findings support the diagnosis of granular cell tumors.

Pathology from the ultrasound-guided core needle biopsy revealed dense, hyalinized stroma with infiltrating aggregates of bland atypical cells exhibiting a histiocytoid appearance, characterized by pink, granular cytoplasm and small nuclei (Figure 4(a)). Positive immunohistochemical (IHC) staining for S-100 (Figure 4(b)), SOX10 (Figure 4(c)), and neuron-specific enolase (NSE) (Figure 4(d)), along with negative pan-keratin staining supported the diagnosis of a granular cell tumor with benign characteristics.

**Figure 5.**
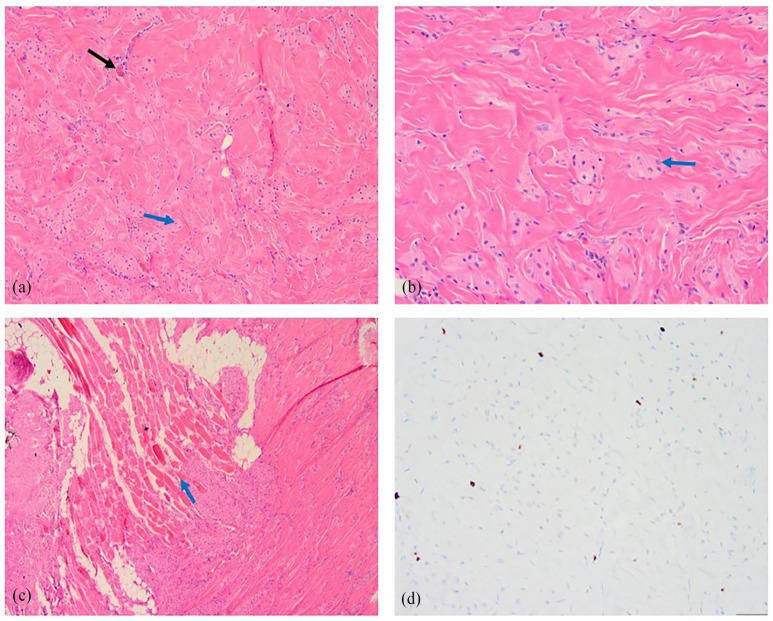
Hematoxylin and eosin staining of the surgically excised breast mass specimen reveals tumor cells (blue arrow) characterized as large and polygonal, with eosinophilic, granular, abundant cytoplasm and centrally placed small and uniform nuclei. Perivascular involvement is observed (black arrow) (a). At higher magnification (×200), tumor cells with indistinct cell borders and forming syncytial groups are evident (blue arrow) (b). Additionally, microscopic evaluation shows tumor cells infiltrating skeletal muscle fibers (blue arrow) (c). Ki67 immunohistochemical staining indicates a low proliferative index, as tumor cell nuclei are negative for this marker (d).

The patient subsequently underwent excision of the right breast mass following ultrasound-guided radar reflector localization. Some of the pectoralis muscle underlying the mass was also resected during the procedure. The final pathology report verified the diagnosis of a granular cell tumor (Figure 5(a) and (b)), with tumor cells infiltrating skeletal muscle fibers (Figure 5(c)). The low tumor proliferation index, combined with the absence of any morphological features suggesting malignancy, supported the benign nature of the disease (Figure 5(d)). All surgical margins were negative.

On follow-up, 2 weeks after the surgery, the patient reported no complaints. The incision was well-healed without any complications. The patient will undergo a contrast-enhanced MRI of the breast in 1 year to assess for any tumor recurrence. In the meantime, the patient underwent a repeat ^18^F-PSMA-PET/CT for the management of his prostate cancer, 2 months after excision of the right breast mass. The scan showed complete resolution of the abnormal radiotracer activity in the right breast.

## Discussion

This case describes the incidental detection of a PSMA-avid mass in the breast of a patient with NCCN very high-risk prostate cancer. The mammogram failed to depict the tumor, as the lesion was located far posteriorly toward the inframammary fold—an area that can be a “blind spot” for mammography, particularly in small breasts. The suspicious features on breast ultrasound prompted further investigation through biopsy, which resulted in the diagnosis of benign granular cell tumor.

PSMA-PET/CT imaging provides highly accurate information for the initial staging of newly diagnosed prostate cancer patients.^1^ However, it is crucial to recognize the causes of PSMA uptake in non-prostatic diseases to ensure treatment planning accuracy and avoid misinterpretation. Apart from its physiological expression, PSMA expression has been observed in other malignant neoplasms, including transitional cell carcinoma of the bladder, hepatocellular carcinoma, non-small cell lung carcinoma, and breast carcinoma.^16^ Breast PSMA uptake has also been reported in association with fibroadenomas, gynecomastia, and PASH.^2–4^ To our knowledge, this case is the first documented instance of intense PSMA uptake in a benign granular cell tumor of the breast.

In patients with breast uptake of PSMA, primary breast malignancies, particularly invasive ductal carcinoma and ductal carcinoma in situ, are key considerations, although metastasis to the breast from prostate cancer has been reported.^6–8^ In our patient, given the medical history of diabetes mellitus, lymphocytic (diabetic) mastopathy is another potential concern.^1^ Other less common entities, such as granular cell tumors of the breast, fibromatosis, and soft tissue sarcoma should also be considered.^17^ The majority of these processes can exhibit suspicious features on mammography and ultrasound, prompting core biopsy for a definitive diagnosis.^5^

The imaging characteristics of granular cell tumors of the breast are nonspecific and often resemble those of carcinoma. Mammography frequently shows hyperdense to isodense focal asymmetry or an irregular mass with obscured or indistinct margins.^10^ Microcalcifications are typically absent.^18^ On breast ultrasound, these tumors commonly appear as irregular, non-parallel masses with heterogeneous echotexture, spiculated margins, and variable posterior shadowing.^10^ On breast MRI, granular cell tumors generally present as masses with spiculated margins, showing isointense to high signal intensity on T2-weighted images, low signal intensity on T1-weighted signal intensity, and variable enhancement patterns.^10^ Previous reports have described variable FDG activity in benign granular cell tumors of subcutaneous tissues and the gastrointestinal tract.^19^ Only one study investigated FDG activity in benign granular cell tumors of the breast, which exhibited no significant radiotracer uptake on PET/CT (SUV_max_ 1.8).^12^ In contrast, quantification of the PSMA uptake in the breast in the present case suggested a malignant tumor, despite benign features described in pathology.

On histopathologic examination, the tumor cells display distinctive granular eosinophilic cytoplasm. Perineural and perivascular involvement is often observed.^20^ IHC staining plays a crucial role in diagnosis, as the tumor usually stains positive for S-100, CD63, CD68, and NSE, indicating its Schwann cell origin.^20^ In our case, positive IHC staining for S-100, SOX10, and NSE supported the diagnosis of granular cell tumor.

Surgical intervention remains the primary treatment approach for granular cell tumors. Given their predominantly benign nature and favorable prognosis, wide local excision is usually sufficient.^21^ Granular cell tumors of the breast have a low risk of local recurrence, even in cases where surgical margins are positive. In the rare instance of malignant granular cell tumors, standard surgical management, including sentinel node biopsy, is recommended, with a limited role for adjuvant therapies such as systemic therapy or radiation therapy.^18^

## Conclusion

We report a case of a man with very high-risk prostate cancer who was incidentally found to have a benign granular cell tumor in the breast, initially detected on PSMA-PET/CT. Knowledge of such extra-prostatic uptake in the breast is essential for interpreting PSMA studies, evaluating subsequent breast imaging, and performing radiologic/pathologic correlation after biopsy.
